# The clinical environment: A facilitator of professional socialisation

**DOI:** 10.4102/hsag.v24i0.1188

**Published:** 2019-02-26

**Authors:** Hester C. de Swardt

**Affiliations:** 1 Department of Health Studies, College of Human Sciences, University of South Africa, South Africa

## Abstract

**Background:**

Competencies of health care workers, including nurses, often do not meet the health needs of populations. The clinical learning environment (CLE) is vital in socialising neophyte student nurses to display the desired competencies. Student nurses are however confronted with challenges, especially in the CLE, during this process.

**Aim:**

This article shares three validated guidelines to support professional nurses and nurse educators in facilitating appropriate professional socialisation of student nurses in the CLE.

**Setting:**

The study was conducted in an 832-bed academic hospital and nine nursing education institutions (NEIs) that offered the nursing programme concerned in a province in South Africa.

**Method:**

A sequential, exploratory, mixed-methods study was conducted and qualitative data were collected from two purposive samples, consisting of seven focus group interviews and field notes. Five themes that emerged from the integrated data guided the instrument design to collect data quantitatively from 277 educators. Experts validated 10 guidelines to a set of criteria, which was developed combining all data.

**Results:**

Qualitative and quantitative research evidenced that the CLE mostly did not support student nurses during professional socialisation. A few role models’ behaviour was noteworthy, while student supervision was inadequate. The CLE was stressful, lacked in resources, marked by uncoordinated student placement, insufficient communication and inadequate preparation of student nurses. This evidence informed the development of the guidelines.

**Conclusions:**

The guidelines were (1) the empowerment of role models through reflective practice, (2) capacity building of professional nurses and nurse educators as clinical supervisors by means of intervention strategies and (3) adopting a multifaceted approach in the creation of a positive CLE. These guidelines could facilitate appropriate professional socialisation of student nurses.

## Introduction and background

According to World Health Organization (2013) competencies, the clinical experience and expectations of health workers, including nurses, often fulfil the health needs of populations poorly. Nurses, the largest number of health care workers, have the potential to address patients’ or clients’ needs (Wong et al. 2015:581). Rispel and Bruce (2015:117) express their concern about nursing shortages, declining interest in the profession and lack of a caring ethos, which affect nursing care delivery. Education and training aim to facilitate appropriately socialised student nurses into the profession, enabling them to provide the care patients or clients need. Fundamentally, the socialisation process deals with the following questions: ‘who am I?’, ‘where do I belong?’ and ‘how do I fit in?’ (Frankland 2010:34). Becoming socialised within a profession entails a continuous process of development of knowledge, skills and professional behaviour to form a professional identity. Several aspects influence this process. The clinical learning environment (CLE) features as a vital component of this process and is irreplaceable in socialising student nurses (Letswalo & Peu 2015:365). The CLE is described as a physical space, characterised by an organisational culture, influenced by psychosocial factors, where communication is important (Flott & Linden 2016:509). Student nurses learn here how nursing is practised, including the implicit values, beliefs and attitudes typical of the profession (Karimi et al. 2014b:53).

The importance of the CLE is evidenced by the ample quantitative (Ford et al. 2016:98; Papastavrou et al. 2016:44) and qualitative (Moagi, Van Rensburg & Maritz 2013:359; Serçekus¸ & Bas¸kale 2016:134) literature on perceptions and experiences of student nurses in the clinical setting. Though important, this setting has some challenges. It is characterised as stressful (Arieli 2013:195), unpredictable (Moagi et al. 2013:259), complex and multidimensional (Flott & Linden 2016:504). In the CLE environment, student nurses learn from what they hear, see and experience. Often student nurses are subjected to uncaring behaviour. Haskins et al. (2014:41) report that patients experience verbal abuse, rudeness and neglect by nurses. Such encounters result in students feeling anxious about their role and being inadequately prepared for the profession (Jack, Hamshire & Chambers 2017:4712). Conversely, Walker et al. (2014:106) report clinical facilitators and professional nurses as being supportive, demonstrating behaviour that students could imitate.

Discussions with undergraduate student nurses revealed that they witnessed and experienced unprofessional behaviour from qualified nurses, which concerned them, and consequently influenced their professional development as student nurses. Professional nurses and educators act as role models in both the clinical and academic environments in shaping student nurses to become professionalised. This information and other similar reports (Haskins et al. 2014:42) resulted in the question: *how could the internalisation of skills, knowledge, values, norms and ethical standards be guided in student nurses to help them become effectively socialised as professional nurses?* This question was answered by developing and validating guidelines. This article focuses on three of the 10 guidelines developed, relating to the clinical environment, with the aim of supporting professional nurses and nurse educators to facilitate appropriate professional socialisation in student nurses.

## Research methods and design

A two-phase, sequential, exploratory, mixed-methods study was undertaken. Firstly, the aim was to explore and describe qualitatively professional nurses’ perceptions of their role in the professional socialisation of student nurses, and student nurses’ experiences of professional socialisation as members of the profession. Secondly, the data and literature were utilised to determine and describe the perceptions of nurse educators on the teaching and facilitation of the professional socialisation of student nurses quantitatively. All evidence and literature informed the development and validation of guidelines to support professional nurses and educators in facilitating appropriate professional socialisation in student nurses.

### Setting, participants and respondents

The study was conducted in an 832-bed academic hospital and all nursing education institutions (NEIs) that offered the nursing programme concerned in a province in South Africa. The hospital treats patients from local clinics and district and regional hospitals, and has a variety of specialised units and health care workers.

During the qualitative phase of the study, two samples were purposively selected: 14 professional nurses and 48 student nurses who were registered for a 4-year nursing programme in general, psychiatric and community nursing, as well as midwifery. The professional nurses supervised the student nurses’ daily nursing activities during their placement for practical experience. The student nurses were from three different NEIs. While student nurses gain practical experience, nurse educators will facilitate the theory and practical learning in cooperation with the clinical area. For the quantitative phase of the study, 277 nurse educators were approached from the nine NEIs responsible for the teaching and facilitation of this nursing programme. The response rate was 46% (*n* = 128). Once the guidelines had been developed, nine of the 12 purposively selected field experts validated them. These experts were from the private and public sectors and represented the clinical and academic or educational settings.

### Data collection

The qualitative data were collected using two focus groups interviews for professional nurses, comprising seven nurses each, and five focus group interviews for student nurses consisting of 7, 10, 10, 10 and 11 participants, respectively. Data saturation guided the samples size. All the interviews were conducted in a quiet boardroom in the hospital during September 2011 and March 2012. Professional nurses’ perceptions were elicited by asking the key question: ‘can you please describe how you perceive or view your role as a professional nurse in the professional socialisation of student nurses?’, while student nurses’ experiences were prompted by asking them, ‘please share your experiences regarding your professional socialisation as a student nurse’. Two different topic guides with possible probing questions were kept at hand; however, the participants’ responses guided the probing and clarification of their answers. Field notes were made to capture non-verbal communication.

A questionnaire was designed, based on the themes and literature from the qualitative phase, to collect quantitative data from nurse educators’ teaching and facilitation strategies of the professional socialisation of student nurses. The questionnaire consisted of six sections, addressing the biographical data, the educator as role model, educator’s facilitation of educational strategies, the educator and the clinical environment, the student nurse and additional comments. The instrument was pretested with respondents who did not form part of the study. The data were collected between June and October 2012 using hard copy questionnaires issued to a liaison person at each NEI. The completed questionnaires were posted in sealed boxes and later collected for analysis.

Qualitative and quantitative data were integrated to develop and validate guidelines that could support professional nurses and educators in the professional socialisation of students.

### Data analysis

After the verbatim transcripts and field notes had been analysed separately by applying Tesch’s (1990) method, the two sets of data were integrated and five themes emerged from this. The themes included the characteristics of a professional nurse, the educator, cultural and gender orientation, values and beliefs related to nursing as a profession and the CLE (De Swardt 2012:91).

Quantitative data were analysed using the SAS Version 9.2 statistical package, which includes descriptive and inferential statistics. The respondents’ self-assessment on the six composite professional socialisation constructs demonstrated a range of less positive to very positive perceptions about their competencies regarding the facilitation and teaching strategies for the professional socialisation of students. These constructs included the characteristics of an educator as perceived by educators themselves and of their colleagues, values and beliefs related to the nursing profession, the educator and the clinical environment, teaching strategies and cultural awareness (De Swardt 2012:121).

Based on the integrated evidence and literature, concluding statements were formulated, from which 10 guidelines were derived, which aimed at supporting professional nurses and educators in the professional socialisation of students. Validation was performed using the following criteria: clarity, comprehensiveness, applicability, adaptability, credibility and validity. Three of the 10 guidelines reported in this article relate to the clinical environment.

### Rigour of the data

Lincoln and Guba’s criteria of trustworthiness, which include credibility, confirmability, dependability, transferability and authenticity, were applied to ensure rigour in the qualitative data. Strategies included prolonged engagement, reflexivity, member-checking, detailed descriptions and keeping meticulous records for auditing purposes.

Validity of the quantitative data was done through content and face validity, while the composite constructs of the questionnaire tested a Cronbach’s alpha of 0.7 to 0.87. Reliability was further ensured through pre-testing of the questionnaire.

### Ethical considerations

The principles of beneficence, respect, human dignity and justice were adhered to throughout the study. After the University of South Africa, Health Studies & Ethics Committee had reviewed and issued an ethics approval certificate (Reference: 3135 840 3), permission was obtained from the various nursing education institutions, academic hospital and the Department of Health. All participants voluntarily participated in the study after signing an informed consent form.

## Results

The qualitative and quantitative data were integrated and are discussed in a narrative manner. Three themes emerged, namely the clinical environment, NEI environment and values and beliefs related to the nursing profession. These guidelines addressed the professional nurse as an exemplary role model and clinical supervisor, as well as the creation of a positive CLE.

### The professional nurse as an exemplary role model

The exemplary role of the professional nurse emerged from the interpretation of qualitative data, which addressed the experiences of student nurses and the perceptions of professional nurses. Student nurses’ experiences were mainly negative regarding the professional nurse as a role model, but they valued those who were admirable role models.

‘You get surprised about the way some of the sisters (professional nurses) are willing to do even the bed pans, ja (yes), I mean the first year’s work or care worker’s work, but I mean it’s still part of the nursing career.’ (L3, female, 4th year)

Student nurses described the role models they were exposed as lacking teamwork, being antagonistic towards students and unethical in their conduct. For instance, professional nurses took food meant for patients:

‘… the registered nurses, the permanent staff, all levels, the permanent staff can’t wait for the food to be dished out because if there’s anything left over … suddenly I have no lunch box and I’m just loading it, apples, eggs, milk, bread, whatever. The patients don’t ever get boiled eggs.’ (M5, female, 3rd year)

Student nurses and professional nurses described noteworthy role models as being knowledgeable, approachable and skilful, excellent communicators, reflective practitioners and having a positive attitude.

Nurse educators’ role model behaviour is important, as 94% (*n* = 112) of educators are responsible for guiding student nurses during their clinical placement. They were regarded as knowledgeable and supportive; however, professional nurses felt some educators did not engage sufficiently with the clinical staff regarding student matters.

Evidence highlighted that the portrayal of skills, knowledge and attitudes could influence student nurses either positively or negatively in their socialisation process.

### The professional nurse as a clinical supervisor

Nurse educators, preceptors, mentors and peers, but mostly professional nurses, supervise student nurses’ nursing interventions in practice to ensure safe nursing care. Educators at the various NEIs indicated that 65% (*n* = 81) of mentors and 49% (*n* = 61) of preceptors assisted with students in practice, while 64% (*n* = 77) used peer mentoring. The role of the preceptor and mentor seemed to be unclear, as professional nurses used these terms interchangeably and felt that this support was limited. Student nurses perceived the role of the professional nurse in their daily nursing activities as more prominent and appreciated the support, but often found themselves being left on their own to perform activities:

‘… we were left alone in the ward, only two students, me and my colleague [*for*] the whole ward … a patient who collapsed there was no one in the ward, all the sisters and staff nurses were on tea and again patient come back [receiving a patient from theatre after a procedure] from theatre no one was there.’ (S9, female, 1st year)

Professional nurses’ limited availability to teach, lack of resources (staff and equipment) and negative attitudes of student nurses hindered clinical supervision.

‘Students are really struggling and nurses (professional nurses) are always busy, you can’t … you’re even afraid to say sister (professional nurse) please help me with something, you can’t go and say sister (professional nurse) can you please help me put in a catheter …’ (S8, female, 3rd year)

Although various forms of support were used in clinical supervision, it seemed that professional nurses were central in the daily nursing activities; however, they faced challenges in fulfilling this role, as student nurses felt alienated in learning new competencies.

### A clinical learning environment

The CLE was characterised by diverse challenges: stressful working conditions, heavy workloads, limited resources, health and safety risks, limited learning opportunities and ineffective communication. Professional nurses realised that the clinical environment should be favourable for learning; however, they were unable to provide the support:

‘No with regards to this teaching. I think we should create an environment or atmosphere, which is conducive to learning. Because most of the time we are busy in the ward and we utilise the student as a workforce, we then forget that they are here to learn.’ (VS5, female, professional nurse)

Entering into the clinical setting could be stressful, as a student nurse explained:

‘… the first days were hard because we were not used to standing up for 12 hours and then the toughest experience was somebody passed away on my first day at the hospital. So it was hard because I didn’t know how to handle it …’ (O10, male, 1st year)

Educators rated the preparation of student nurses for their first clinical exposure slightly more negatively compared to other items related to the construct, ‘the educator and the clinical field’s challenges’. Of the 112 educators who responded, 66 (59%) indicated in a follow-up open question that simulation is mostly utilised, while only 14 (13%) mentioned discussion and debriefing as preparation methods.

Student nurses also felt frustrated about not receiving sufficient learning opportunities, as one explained:

‘… the sisters told me that you just gonna do bed pans only and if the bell rings just go to the person that’s all you going to do and I never got a chance to do my procedures but then I went to another hospitals then I did my procedures.’ (NM13, female, 1st year)

In addition, educators stated that in some instances clinical facilities are overcrowded with students, which further limit learning opportunities. About 82% (*n* = 103) of educators affirmed that the minimum 4000 h prescribed by the South African Nursing Council received preference above the learning outcomes of students, prohibiting further learning.

Communication between the NEIs and the clinical field about student matters is essential; however, student nurses exploited this lack of communication, as professional nurses perceived it as their authority being undermined.

The multiplicity and challenges of CLE are evident from the educators’, professional nurses’ and students’ reports.

Using the above evidence and literature, the concluding statements below, with recommendations, were formulated as guidelines (Table 1).

**TABLE 1:** Summarised concluding statements to three guidelines.

Theme	Category	Concluding statements	Guideline	Recommendations
Clinical environment	The professional nurse as exemplary role model	Students nurses’ experiences regarding professional nurses as role models were mostly negative, but they respected positive role modelling behaviour Educators were regarded as worthy role models Noteworthy role models were described	The professional nurse as exemplary role model	Empowerment of role models
	The professional nurse as clinical supervisor	Professional nurses did not comply with their clinical supervisor’s role as expected Constraints such as workload, resources and attitudes hindered the professional nurses’ clinical supervisors’ role Preceptor and mentoring roles were ill-defined; educators reported providing support but felt it was limited	The professional nurse as clinical supervisor	Capacity building of the professional nurse and nurse educator as clinical supervisor
	A positive clinical learning environment	Clinical learning environment (CLE) was complex and described as stressful, with increased workloads, inadequate resources, health and safety risks, limited learning opportunities and ineffective communication Simulation was mainly used to prepare student nurses for their first clinical exposure Learning outcomes did not feature as a priority, as the minimum hour requirement received preference; learning opportunities were mismatched with student nurses’ needs	A positive clinical learning environment	Creating a positive clinical learning environment

## Discussion

Many studies have been conducted on the importance of role models in the development of a professional identity (Jack et al. 2017:4712; Tiwaken, Caranto & Jose David 2015:73). Professional competencies such as ethics and patient-centeredness are mostly learnt through observation of role models’ behaviour (Karimi et al. 2014a:6). Although professional nurses were aware of the influence of role modelling on students, student nurses experienced that professional nurses predominantly modelled negative behaviour. Negative role modelling affects morale and the perception of nursing (Walker et al. 2014:106), whereas positive role modelling could enhance the socialisation process and the construction of a professional identity (Jack et al. 2017:4712). Student nurses appreciated role models that could teach and were willing to participate in all nursing activities. Educators seemed to fulfil their role modelling responsibility as perceived by student nurses and themselves; however, their communication with stakeholders on student matters was ineffective. Collaboration between educators and clinical practice is essential to facilitate integrated learning (Bjørk et al. 2014:2964).

To ensure that student nurses’ learning outcomes are realised, successful clinical supervision is essential (Hall-Lord, Theander & Athlin 2013:507). The consequences of unmet clinical learning outcomes could lead to a generation of nurses entering the workforce with limited knowledge and skills and inappropriate attitudes to provide the desired care for their patients or clients. Factors such as professional nurses’ competency, workload, resources and organised clinical supervision structure are elements that affect the clinical supervision role of the professional nurses and educators. Numerous studies globally report on scarce resources, for example insufficient supervisory structures and staff and equipment shortages that have an impact on student learning (Clements et al. 2016:20; Ford et al. 2016:98; Haskins et al. 2014:42). Student nurses value professional nurses fulfilling their clinical supervision role, which is supported by Jack et al.’s (2017:4707) study conducted in the UK. Student nurses feel exposed and discontented if no or limited clinical supervision takes place.

The complexity and interrelationships of the CLE make intentional teaching approaches in this environment necessary for the appropriate professional socialisation of student nurses. Student nurses, professional nurses and educators expressed their concern about factors contributing to the undesirable professional socialisation of students. Stressful first-time encounters in practice (reality shock) caused students to feel overwhelmed and anxious despite educators using simulation to prepare them. Although students could be prepared for the clinical field, they are still ‘shocked’ and stressed when exposed to a real situation partly because of unmet expectations (Al Awaisi, Cooke & Pryjmachuk 2015:1733). Frustration, disrespect and exposure to unethical practices challenge the desired professional socialisation of student nurses (Sabatino et al. 2015:317). The unpredictable stressful clinical environment influences learning outcomes in terms of opportunities, workload, health and safety risks, and support. Uncoordinated student placement, hour requirements and inadequate communication between the clinical facilities and NEIs hinder an appropriate socialisation process. A well-planned placement schedule, continuous communication and building professional relationships (Ford et al. 2016:102) are essential for learning that matches the students’ needs.

## Guidelines

The three guidelines, ‘the professional nurse as exemplary role model’, ‘the professional nurse as clinical supervisor’ and ‘a positive clinical learning environment’, included the empowerment of role models, the capacity building of the professional nurse and nurse educator as clinical supervisor and the creation of a positive CLE.

### Empowerment of role models

Empowering of role models to model nursing values, which include human dignity, integrity, autonomy, altruism and social justice, might facilitate appropriate professional socialisation in student nurses. The importance of role modelling is emphasised as Sanderse (2013:39) postulates that moral education of students occurs by means of role modelling. Moral education focuses on value clarification, cognitive development, care ethics and character education (Sanderse 2013:28). Enabling role models to become reflective about their own practice, explaining their actions and value orientation, could assist in moral education. Reflection or reflective practice elicits considerable interest in nursing education as a method to develop professional nursing (Karimi et al. 2017:1). Teaching reflection does not occur naturally; it requires support and guidance. It is a process of self-discovery, learning from past events to gain better understanding in dealing successfully with similar events in future (Pretorius 2016:241). Self-awareness, professional growth and behaviour are facilitated by implementing, for example, reflective writing (Naber & Markley 2017:1), reflective group discussions and storytelling. Creating a reflective practitioner enhances knowledge and changes assumptions, values and beliefs, which informs clinical practice (Miraglia & Asselin 2015:71) and could consequently empower the professional nurse to model the core values of nursing.

### Capacity building of professional nurse and nurse educator as clinical supervisor

Clinical supervision aims to be supportive in developing safe and reflective nurse practitioners (Maplethorpe, Dixon & Rush 2014:183). These authors proposed a capacity building strategy by means of a 4-day training course of clinical supervision, using a humanistic clinical supervision approach. They found in this UK study that mental health users (professional nurses) were more sensitised to clinical supervision and their role preparation. They suggested continual support to professional nurses, as it would add value to student nurses’ learning experiences. A similar capacity building intervention approach in Australia yielded a favourable psychosocial CLE for student nurses (Henderson et al. 2010:180). An experienced educator or researcher conducted this intervention through discussions on best evidence practices with registered nurses during a 6-week period. It is argued that this intervention could be sustainable if it is embedded and accepted as part of the organisation’s values, norms and behaviours. Another capacity building strategy, also implemented in Australia, includes the Art of Clinical Supervision (ACS) programme, which focuses on rewarding positive role modelling, having discussions about topics that are aligned with the group’s needs and sharing stories about positive and negative incidents (Russell, Alliex & Gluyas 2016:10). The ACS programme has facilitated positive attitudes to clinical supervision in professional nurses.

Scarce resources are a challenge, as is evident in the current study. The Clinical Nursing Leadership Learning and Action Process Model (Jumaa & Rendal 2007:114) approach was successfully implemented in Nigeria to address scarcity of human, time, equipment, information, material and money resources. The key questions that guided this model were the following: ‘what would one like to do?’, ‘what can be done?’, ‘what could be done?’ and ‘what must be done?’ This model assists professional nurses to apply theory into practice, be effective team leaders and manage the environment efficiently, which would enable them to implement their clinical supervising function.

The current study revealed that clinical supervisory structures did not seem to be well structured, which led to miscommunication between NEIs and the clinical field. This view is supported by Kgafele, Coetzee and Heyns (2015:S238), who suggest that clear communication regarding learning outcomes is essential. Close collaboration between preceptors and educators, complementing each role, is recommended to maximise the academic and clinical learning of student nurses (Lawal et al. 2016:37).

### Creating a positive clinical learning environment

Student nurses should be prepared to deal with the challenging clinical environment and it should be emphasised that they are jointly responsible and accountable for their own learning, for instance utilising all possible learning opportunities (Ford et al. 2016:101). Professional nurses should give student nurses responsibility, as this is perceived as having trust and being valued (Sandvik, Eriksson & Hilli 2015:69). It is important that purposeful learning opportunities are assigned, which could be realised if professional nurses are aware of the intended learning outcomes of each year level. Learning is hindered when student nurses are stressed, and this can be best addressed through debriefing and reflection, as these strategies lead to reframing and redirection, and ultimately to resilience (O’Mara et al. 2014:211).

Support to student nurses is related to having trusting professional relationships (Walker et al. 2014:109). Student nurses thrive when treated with respect (Ford et al. 2016:102). Conversely, when disrespect and hostility are experienced, it results in anxiety and learning is restricted, as is evident from the current study. Positive relationships support the development of a desirable professional self-concept (Clements et al. 2016:25), while negative relationships may reinforce inappropriate professional identity formation.

Excessive workloads and lack of resources are also reported in other studies (Mwale & Kalawa 2016:5; Ó Lúanaigh 2015:452). This challenge is a reality that requires creativity. Hayes et al. (2012:889) found that staff could manage it as long as they have team support. In addition, student nurses’ professional socialisation is positively influenced by being included as team members. They experience a sense of belonging when good teamwork occurs (Warhurst 2011:880).

Coordinated optimal use of clinical facilities requires planning, partnership with all stakeholders (Doyle et al. 2017:31) and adequate preparation. Grace and O’ Neil (2014:302) successfully piloted a better Prepared Better Placement resource online in Australia as a tool to prepare health care students, including nurses, for their clinical placement. This tool provides current information, assisting students in setting goals and exposing them to case studies similar to cases they might possibly encounter.

Creating a positive CLE involves a multifaceted approach, in which sound communication, leadership and teaching strategies focused on the student nurse are vital.

## Conclusion

This study utilised a sequential, exploratory, mixed-methods design to develop and validate guidelines, specifically in creating a positive clinical environment to support the development of a desirable professional identity. Qualitative and quantitative evidence revealed that the CLE mostly did not support student nurses in their professional socialisation. Student nurses witnessed role models whose behaviour was questionable, while they admired those professional nurses and educators who were noteworthy and supportive. Clinical supervision of student nurses was inadequate in some instances because of the workload, attitudes and unstructured clinical support. The CLE was characterised as stressful, lacking in resources, using uncoordinated student placement with the emphasis on hour requirements, insufficient communication and inadequate preparation of student nurses for the clinical field. The professional socialisation of student nurses was influenced either positively or negatively by these factors. Three guidelines were developed and validated to empower role models through creating reflective practitioners, capacity building of professional nurses through using intervention models and supportive programmes. All stakeholders can collectively create a positive CLE by forming trusting relationships, facilitating teamwork, communicating, planning and applying effective leadership. These recommendations regarding the CLE require a deliberate multidimensional approach to facilitate appropriate professional socialisation in student nurses.
